# Reprogramming the chromain landscape at eukaryotic regulatory elements

**DOI:** 10.1186/1756-8935-6-S1-O20

**Published:** 2013-03-18

**Authors:** Gordon L Hager

**Affiliations:** 1 Lv of Receptor Biology & Gene Expression, NIC/NIH, Bethesda, MD, 20892, USA

## 

Eukaryotic transcription factors (TFs) regulate gene expression by interacting with chromatinized DNA response elements (REs). Access to these elements is dramatically restricted by chromatin organization, and modification of the nucleoprotein structure to allow factor binding is emerging as a key feature of cell selective gene regulation [1,2,3,4]. These processes are highly dynamic, often with oscillatory or cyclical components operating on multiple time scales. Furthermore, many regulatory elements are located at great distance from target genes; long range interactions must play a significant, possibly dominant, role in gene regulation [5,6]. We will discuss an integrated dynamic model for regulatory element function on multiple scales.
